# A Protocol on Using the RE-AIM Framework in the Process Evaluation of the Primary Health Integrated Care Project for Four Chronic Conditions in Kenya

**DOI:** 10.3389/fpubh.2021.781377

**Published:** 2022-01-12

**Authors:** Violet Naanyu, Hillary Koros, Beryl Maritim, Jemima Kamano, Kenneth Too, Obed Limo, Gladwell Gathecha

**Affiliations:** ^1^Department of Psychology Sociology and Anthropology, School of Arts, Moi University, Eldoret, Kenya; ^2^The Academic Model Providing Access to Healthcare (AMPATH), Eldoret, Kenya; ^3^Department of Medicine, College of Health Sciences, Moi University, Eldoret, Kenya; ^4^Department of Non-communicable Diseases, Ministry of Health Kenya, Nairobi, Kenya

**Keywords:** RE-AIM evaluation framework, Kenya, PIC4C model, process evaluation, primary health care (PHC)

## Abstract

**Background:** There has been a rapid increase in morbidity and mortality arising from non-communicable diseases (NCDs). The Academic Model Providing Access to Healthcare (AMPATH) program has established a chronic disease management program in collaboration with the Ministry of Health (MoH) in Kenya at over 150 health facilities in western Kenya. The primary health integrated care for chronic (PIC4C) disease project seeks to deliver preventive, promotive, and curative care for diabetes, hypertension, cervical and breast cancers at the primary health care level. We apply the RE-AIM framework to conduct a process evaluation of the integrated PIC4C model. This paper describes the protocol we are using in the PIC4C process evaluation planning and activities.

**Methods and Analysis:** This evaluation utilizes clinic reports as well as primary data collected in two waves. Using mixed methods (secondary data, observation, semi-structured interviews, and focus group discussions), the process evaluation assesses the reach, effectiveness, adoption, implementation and maintenance of the PIC4C model in Busia and Trans Nzoia Kenya. The evaluation captures the PIC4C process, experiences of implementers and users, and the wishes of those using the PIC4C services. We will analyse our data across the RE-AIM dimensions using descriptive statistics and two-sample *t*-test to compare the mean scores for baseline and end line. Qualitative data will be analyzed thematically.

**Discussion:** The process evaluation of the PIC4C model in Kenya allows implementers and users to reflect and question its implementation, uptake and maintenance. Our experiences thus far suggest practicable strategies to facilitate primary health care can benefit extensively from deliberate process evaluation of the programs undertaken. Furthermore, integrating the RE-AIM framework in the process evaluation of health programs is valuable due to its pragmatic and reporting usefulness.

## Introduction

Sub-Saharan Africa is home to 11% of the world's population yet it accounts for the largest proportion of the global chronic disease burden (1). In Kenya, cardiovascular diseases and cancers alone are the highest contributors to mortality accounting for 8.6 and 7% of mortality in the country, respectively (2). Consequently, high healthcare expenses and reduced productivity associated with chronic diseases continue to impose huge strain on households and developing economies in low and middle-income countries, slowing economic and social development (3). This underscores the need for well-designed chronic disease management interventions to improve service delivery and ultimately, improve health outcomes (4, 5). A large body of evidence supporting the integration of chronic disease care has been demonstrated by various interventions globally (4, 6). Indeed, innovative integrated care primary care models have been designed and piloted based on existing evidence for early diagnosis and management of chronic diseases yielding better health outcomes (7, 8).

Despite the evidence to support integration of healthcare, replication and uptake of innovative primary healthcare programs has been slow and inequitable (9, 10). The Kenya Ministry of Health (MoH) in partnership with the World Bank and Access Accelerated piloted an integrated care model for hypertension, diabetes, breast, and cervical cancer at the primary health level known as PIC4C (11). The PIC4C model was designed to address four specific objectives: (1) To explore perceived barriers and facilitators to prevention and management of select NCDs at the primary health care level; (2) To describe the process of implementation of the integrated care model for the four conditions; (3) To evaluate the effectiveness of the integrated care model; and (4) To estimate the incremental cost and budget impact of scaling up the model. The integrated model provides services across the tiers/levels of care.

The Kenyan health system has six levels of care. They include level 1, community services; level 2, dispensaries and clinics; level 3, health centers and maternity and nursing homes; level 4, Sub-County hospitals and medium-sized private hospitals; level 5, County referral hospitals and large private hospitals; and level 6, national referral hospitals and large private teaching hospitals (5). The objective of this protocol is therefore to describe a process evaluation on the effectiveness of the integrated chronic disease model in western Kenya using the RE-AIM framework in order to inform MoH policy and scale up.

Process evaluations are increasingly being used by researchers as roadmaps to unpack the reasons why programs succeed or fail by providing information on the context, underlying mechanisms and outcomes (1, 8, 12). Translating the growing field of knowledge from population health interventions to policy and practice depends on a research to implementation bridge facilitated by accompanying planning and evaluation models like RE-AIM (5). RE-AIM framework is widely used in planning and evaluation particularly of research programs with growing application in non-research fields. The framework is particularly useful in contributing to the understanding of the scale up of complex community programs such as PIC4C by evaluating the intervention's Reach, Effectiveness, Adoption, Implementation and Maintenance (11).

The RE-AIM framework has been in use for over 20 years and guides researchers in the design, implementation and evaluation of implementation research and programs with the primary objective of making findings more generalizable (10, 13). RE-AIM is an acronym for the five dimensions explored in the use of the framework (Reach, Effectiveness, Adoption, Implementation and Maintenance) and are further categorized into individual levels and setting level outcomes to consider in assessment (5)]. At the population level, we have Reach (R), Effectiveness (E) and Maintenance (M) while system level considers Adoption (A), Implementation (I) and Maintenance (M) by the program agents. The RE-AIM framework has been lauded for its innate ability to provide a nuanced assessment of the barriers and facilitators to successful implementation of intervention.

One of the key strengths of the RE-AIM framework is its ability to lend itself to adaptation in different settings, offering a practical approach to program evaluation in low resource settings. While the five dimensions laid out form a comprehensive perspective, evaluations can adopt the dimensions practical to the intervention, setting, and availability of data and resources (5). Thus, the framework has been used to evaluate community and clinical programs across the globe and in Kenya (10). This paper describes the use of the REAIM framework to evaluate the PIC4C integrated chronic care model for hypertension, diabetes, cervical and breast cancers within primary health care setting in Busia and Trans Nzoia counties of western Kenya.

## Methods and Analysis

The PIC4C model delivers preventive, promotive, and curative care for diabetes, hypertension, cervical and breast cancers at the primary health care level. It covers 40 and 33 health facilities in Busia and Trans Nzoia, respectively. Trans Nzoia County lies on the eastern side of Mount Elgon, in western Kenya. As per the 2009 census, Trans Nzoia County has a population of about 818,759 people, and 50% are male. There is one County referral hospital, five Sub-County hospitals, and seven health centers. Busia County is situated in the western part of Kenya and borders Uganda. The County covers an area of 1,694.5 square kilometers and has a population of 953,337 and 47.8% are males. Busia has one County referral hospital, six Sub-County hospitals, and fourteen health centers (14).

Primary health facilities are the first level of contact between patients and the health system. They include Health Centers and Dispensaries. They provide ambulatory health services, which are generally preventive, and curative services mostly adapted to local needs. Common services provided as prioritized by the Kenyan government include education on health problems and how to prevent them, nutrition, maternal child healthcare, family planning, basic sanitation, immunization, treatment of common diseases and injuries, and provision of basic medication. Additionally, select primary health care facilities address dental health, mental health, HIV AIDS, and primary eye care. None of the health facilities involved in PIC4C included routine preventive and treatment care for cancers, hypertension, or diabetes.

The overarching goal of the evaluation of the PIC4C project is to document the reach, effectiveness, adoption, implementation and sustainability of the integrated chronic care model with the aim of informing the ministry of health policy and scale up of the model. The PIC4C process evaluation uses mixed methods to ensure all aspects of the RE-AIM are well addressed. Supplementary Files 1–6 show the surveys, in-depth interviews and FGDs that were used to collect information from patients, health care workers, and decision makers.

### PIC4C Process Evaluation Design

This paper describes the protocol we are using in the PIC4C process evaluation planning and activities. Data will be collected in two waves, 18 months apart, using mixed methods: secondary data, semi-structured interviews (SSIs), observation and focus group discussions (FGDs). The secondary data will involve analyses of routinely collected PIC4C care and project activity data including daily registers and the monthly reports. A SSI is a qualitative data generation method, which allows for a natural dialogue around a topic of interest. A predetermined list of questions is used to develop an interview schedule which is used to guide the discussion. There will be 48 SSI sessions involving County leaders including the CEC and Director Health, Non-communicable Diseases Coordinator). While the socio-demographics data will be collected using a structured questionnaire, the rest of the questions will be open-ended.

The observation method generates qualitative data on specific activities of interest. In each County (Table 1), we will conduct 16 patient reception and vital signs assessments at health facility level. During these sessions, we will observe how patients are received upon arrival at the health clinic and how vital signs are assessed. The education and screening observation assessment will be done at the community level only during the community screening services led by the Community Health Promoters (CHPs). We aim to make 8 observations in each County. The FGD is a qualitative data collection method involving 6–12 participants with a trained moderator to guide the discussions around a particular topic. It is useful to gain a shared ideas and opinions among participants. A total of 20 FGD sessions will be held as follows (Table 2): Clients/patients 8 FGDs; health care providers (HCPs) 8 FGDs; community health promoters (CHPs) and community health volunteers (CHVs) 4 FGDs.

**Table 1:** Recruitment for Semi Structured Interviews (SSIs).

**Participants**	**Position/cadre/Sub-County**	**Composition by gender**	**Number of SSIs**	
			**Busia**	**Trans Nzoia**
County level	CEC member	Mixed gender	1	1
	Director of Health – NCDs	Mixed gender	1	1
	NCD focal person	Mixed gender	1	1
Sub-County level (Medical Officer, Nurse In-charge, RH Coordinator, HRIO, Pharmacist)		Mixed gender	2	2
		Mixed gender	2	2
		Mixed gender	2	2
		Mixed gender	2	2
		Mixed gender	2	X
		Mixed gender	2	X
		Mixed gender	2	X
Health Facility In-Charge	County level	Mixed gender	1	1
	Sub-County level	Mixed gender	3	3
	Health Center level	Mixed gender	3	3
	Dispensary level	Mixed gender	2	2
	Total		27	21

*CEC, County Executive Committee member for Health; HRIO, Health Records Information Officer; NCD, Non-Communicable Diseases; RH, Reproductive Health*.

**Table 2:** Recruitment for Focus Group Discussions (FGDs).

**Participants**	**Group composition**	**Composition by gender**	**Number of FGDs**	
			**Busia**	**Trans Nzoia**
Patients with either of the NCDs	Diabetes	Mixed gender	1	1
	Hypertension	Mixed gender	1	1
	Breast cancer	Female	1	1
	Cervical cancer	Female	1	1
Health care providers	Trainees/mentees	Mixed gender	1	1
		Mixed gender	1	1
		Mixed gender	1	1
		Mixed gender	1	1
CHPs	CHPs	Mixed gender	1	1
CHVs	CHVs	Mixed gender	1	1
Total	10	10

*CHP, Community Health Volunteer; CHV, Community Health Worker; NCD, Non-communicable Disease; RH, Reproductive Health*.

The RE-AIM framework will be used and we aim to interview the same study participants at each time point and recruit additional participants where appropriate. In Table 3, the RE-AIM dimensions are applied to specific aspects of the PIC4C project. Indicators to show expected activities and/or outcomes are described. Data sources for required information and relevant data collection methods are also provided. For the reach, absolute number and characteristics of targets of the intervention are described. The absolute number of patients and trainees/mentees engaged will be reported, including their specific characteristics. The reach will also capture the number of people educated, screened, linked, treated and retained for each condition will be captured. Effectiveness covers the impact of the PIC4C interventions with special attention to numbers linked, treated and retained for each condition, and client feedback on the PIC4C services. The following changes will be expected among patients: a drop of 10 systolic blood pressure (SBP) or 5 diastolic blood pressure (DBP); % of diabetes mellitus (DM) patients getting to <8%, or mean drop of 2%; % of positive screened treated for pre- cancer lesions or linked for cancer treatment or those treated for pre-cancer lesions who are cancer free at one year follow up. For those with breast cancer, % of breast lumps biopsied and % of cytology positive linked to care would be of interest. For the trainees/mentees, the evaluation will reveal their change in knowledge, skills, and confidence in providing service for the four conditions, as well as trainees feedback on the PIC4C project. Regarding adoption, the evaluation focusses on patients' adherence to clinic visits and their NHIF uptake. At the health facility, adoption is seen at four levels: (1) Percentage of MoH trained staff offering care in the training area, (2) level of implementation of NHIF, (3) Use of PIC4C initiated information technology systems, and (4) Introduction of MoH tools and % using them. Lastly, at the County level, the number of budgets that include PIC4C strategies for any of the four conditions will be reported.

**Table 3:** PIC4C process evaluation indicators and data sources.

**RE-AIM dimension**	**Definition**	**Indicator**	**Data sources**
Reach	Proportions and numbers of PIC4C participants	- Absolute number of patients and trainees/mentees engaged - Absolute number of people educated, screened, linked, treated and retained for each condition - Characteristics of CHVs, CHPs, Patients and Providers who took part in PIC4C programs	Monthly PIC4C reports
Effectiveness	PIC4C impact on important outcomes	**a. Client level** - Drop in SBP and DBP - Controlled blood sugar levels - For CaCx, % of positive screened treated for pre-Ca lesions, or linked for Ca treatment, and finally % of treated for pre-Ca lesions who are Ca free at 1 year follow up - For Ca breast, % of breast lumps biopsied and % of cytology positive linked to care - Patients linked, treated and retained for each condition - Time of diagnosis to treatment - Client feedback on services **b. Trainees/Mentees level** - Change in level of knowledge, skills, and confidence in provision of services - Trainee feedback	- Monthly PIC4C reports - FGDs and SSIs with patients, providers, CHVs, and CHPs
Adoption	Institutional and individual level willingness to initiate PIC4C programs	**a. Client level** - Adherence to clinic visits - NHIF uptake **b. Health facility level** - % of MoH trained staff offering care in the training area - % of facilities run by MoH staff fully i able to run without mentors ii with only occasional mentorship - Implementation of NHIF - Use of PIC4C initiated information technology systems[-] - Introduction of MoH tools and % using them **c. County level** - Number of County strategies/budgets including PIC4C strategies for any of the four conditions	- Monthly PIC4C reports - FGDs & SSIs with patients, providers, CHVs, and CHPs - SSIs with County officials and opinion leaders
Implementation	Fidelity to elements of the PIC4C protocol, and clients' use of the interventions	**a. Health facility and community programs** - Fidelity to the various elements of PIC4C protocol (consistency of delivery as intended, and at planned time and cost) - Level of completeness of program set up **b. Client level** - Client use of PIC4C intervention strategies	- Monthly PIC4C reports for implementation processes and resources used - FGDs and SSIs with patients, providers, CHVs, and CHPs
Maintenance	The extent to which PIC4C becomes institutionalized and long-term effects of the interventions	**a. Client level** - Adherence and retention of patients in care - Loss to follow up - NHIF maintenance - Accessibility to drugs and ability to meet care costs **b. Health system level** - Level of institutionalization of revolving fund pharmacy and its maintenance - Level of institutionalization of information technology systems and their maintenance - Percentage of facilities willing and able to continue with PIC4C programs **c. County level** - Uptake of clinical staff from PIC4C into County healthcare - Institutionalization of CHV payment - Percentage of Sub-County Health Management Committees (SCHMT) able to continue activities	- Monthly PIC4C reports - FGDs & SSIs with patients providers, CHVs, and CHPs - SSIs with health facility in-charges - SSIs with SCHMT members

*CHV, Community health volunteer; CHP, Community Health Volunteer; FGD, Focus group discussion; MoH, Ministry of Health; NHIF, National Health Insurance Fund; PIC4C, Primary Health Integrated Care for Chronic (PIC4C) disease; SCHMT, Sub-county Health Management Committee; SSI, Semi structured interview; SBP, Systolic Blood Pressure; DBP, Diastolic Blood Pressure; Ca, Cancer; CaCx, Cancer of the cervix*.

Implementation focusses on the extent to which the PIC4C programs have been delivered as intended and appreciates any deviations/adaptations applied. This includes fidelity to the PIC4C activities, timing and costs. It also captures level of completeness and utilization of PIC4C interventions. The final dimension of the RE-AIM is maintenance, which considers sustained effectiveness of the PIC4C at the individual level and the sustained delivery at the institutional level in Busia and Trans Nzoia.

### Study Participants

The study participants will be clients, health care workers, and decision makers. The clients will include patients with diabetes, hypertension, cervical and breast cancers. Health care workers will include Community Health Promoters (CHPs), Community Health Volunteers (CHVs) and health care providers (being trainees and mentees). Decision makers are facility in-charges, Sub-county and County leaders. Table 4 provides the study participants and the tools that will be used to interview them.

**Table 4:** PIC4C process evaluation study participants, tools and analysis plan.

**Tools**	**Content/description**	**Number of sessions/participants**	
		**Busia**	**Trans Nzoia**
Patient reception and vital signs assessment:	Registration process and availability of records Equipment availability Assessment of the BPs, BMIs and RBS Clinical care Comparing what is happening vs. ideal Analysis plan: Proportions, Mean percentage frequencies and *t*-test	8^*^ 16^**^	8^*^ 16^**^
Education and screening observation checklist	Duration of screening session Content of the education session for the health care providers Screening equipment availability Measurements taking process Referral process Analysis plan: Proportions, Mean percentage frequencies and *t*-test	8	8
Written test/Random Knowledge test (CHVs/CHPs)	CHP Written test- Multiple choice; Content: Diabetes Hypertension Breast cancer Cervical cancer Analysis plan: Proportions, Mean percentage frequencies and *t*-test	16	16
Written test/Random Knowledge test (Clinicians)	Stratified based on facility type Analysis plan: Proportions, Mean percentage frequencies and *t*-test	8	8
Patient Self-Report/feedback forms	Patient experience with care and treatment Adherence Usefulness of the services provided (Likert scale) Analysis plan: Proportions, Mean percentage frequencies and *t*-test	384	384
SSIs with facility, Sub-County and County leaders	County leaders (CEC and Director Health, NCD Coordinator); Focused on Adoption and Maintenance Sub-County leaders (Medical Officers, Nurses, RH coordinators, Pharmacists and HRIO); Focused on Adoption and Maintenance Facility In-Charge (9 per County); Focused on Adoption and Maintenance Analysis plan: Thematic analysis of transcripts	24	24
FGDs with clients	Questions focused on Effectiveness of the PIC4C model (does it achieve the intended outcome and impact? – Adherence, Behavior change, Utilization of services at the primary level facility) Compare patient level opinion to objective effectiveness (change in BP, RBS values) Analysis plan: Thematic analysis of transcripts	4	4
FGDs with Health care providers (HCP)	Target: Trainees and Mentees Focus: Effectiveness, adoption and maintenance Stratification by cadre Analysis plan: Thematic analysis of transcripts	4	4
FGDs with CHPs/CHVs	4 sessions; 2 CHPs and 2 CHVs sessions Focus: Effectiveness, adoption and maintenance Analysis plan: Thematic analysis of transcripts	2	2

*BPs, Blood Pressure; BMI, Body Mass Index; CEC, Chief Executive Officer for Health; CHV, Community health volunteer; CHP, Community Health Volunteer; HRIO, Health Records Information Officer; NCD, Non-Communicable Diseases; PIC4C, Primary Health Integrated Care for Chronic (PIC4C) Disease; RBS, Rapid Blood Sugars; RH, Reproductive Health*.

* *Community level*;

** *Facility level*.

The FGD and the SSI participants will be selected purposefully in each county based on their health condition (e.g., diabetic or hypertensive patients). The health care providers and decision makers will be selected in each county based on their cadre and level of their facility; County, Sub-County, Health Center or Dispensary. In addition, the sampling for the observations on patient reception and vital signs assessment in each County will be based on the facility level to ensure that each level is represented. The education and screening observation will be based on the number of community health promoters who are leading these sessions. The patient self-report surveys will be based on a sample size calculated to ensure a representation of the patients receiving care and treatment services for the four conditions under the PIC4C project in each of the Counties.

### Recruitment of Study Participants

Trained research assistants will recruit all study participants using predetermined inclusion criteria. For health care providers, they would have to be working in the designated County, Sub-County or health facility for at least 6 months, and be able to speak in English or Kiswahili.

We aim to interview at least 3 individuals at the County level leadership. At Sub-County level, we aim to interview at least 9 individuals including the non-communicable disease (NCD) focal persons, Reproductive Health Coordinators, Medical Officers, Nursing officers, Health Records Officers and Pharmacists. At the facility level, we aim to interview at least 9 healthcare facility in-charges. We aim to have a mixed gender representation at all the recruitment levels (Table 1).

For the FGDs (Table 2), the first categories involve clients. We will engage patients who should be mentally stable, living with any of the four NCDs (Diabetes, hypertension, breast cancer, and cervical cancer), aged between 18 and 60 years and be able to speak English or Kiswahili. For the HCP FGDs, participants should be working in the designated facility for at least 6 months. For the CHP and CHVs FGD participants, they should have worked for the PIC4C project for at least 6 months, aged between 18 and 60 years and able to speak English or Kiswahili. We aim to recruit 8–12 participants per FGD session. There will be 8 FGDs with patients (4 per County) and the FGDs on diabetes and hypertension will have mixed gender, while those addressing breast and cervical cancer will have females only. The 8 FGDs for the HCPs (4 per county) will be composed of the trainees and mentees for the NCD conditions and they will be from different cadres (see Table 2).

Prior to beginning work in each county and each facility, approvals will be sought accordingly. A PIC4C local contact at the facilities and a research team member will meet with the relevant health facility leadership and discuss the process evaluation of the PIC4C project. At each health facility and prior to conducting consent, one of the RAs/research team members will recruit relevant HCPs, CHPs, and CHVs into the study. The PIC4C RA will approach the health worker individually, share with them the purpose of the data collection session, and confirm interest in participating. For those interested, the RA will schedule the session at a time convenient to the participant. For the patients, they will be identified from the PIC4C database based on their condition. Potential participants will be called and recruited by telephone 1–2 weeks before the FGD sessions that will be held in the nearest health facility and at times that are convenient to participants.

### Data Collection

The study will use both routinely collected clinic data (e.g., daily registers and the monthly registers/reports) and data that will be specifically collected during the two waves. In wave one, the tools for data collection include: (1) Patient reception and vital signs' assessment checklist (Observation process mapping); (2) Education and screening observation checklist; (3) Written test/Random knowledge test for CHVs, CHPs and Clinicians; (4) Patient self-report/feedback forms; and (5) Health facility questionnaire. In wave two, all wave 1 tools will be administered. In addition, there will be SSIs with facility, Sub-County and county leaders; and FGDs with clients, HCPs, CHPs, and CHVs.

Questionnaires will be administered at the facility, at baseline and end line, to compare availability of select drugs for hypertension and diabetes. Data will also capture the time of diagnosis to treatment for diabetes and hypertension care. For lost to follow-up, we shall use the return to clinic date. For those on medication, we shall check if they have defaulted 90 days from the last return to clinic. We shall also check for those on lifestyle modification.

Trained research assistants will facilitate semi-structured interviews (SSIs), FGDs and observations. They will ensure all participants provide written consent before they participate in the evaluation. They will observe how patients are received, do process mapping during care, and report on how vital signs are assessed. They will also observe the education and screening activities. Regarding the FGDs, each session will be facilitated by a moderator and a scribe. The scribe will help the moderator by taking notes during the discussions. All sessions will be audio-recorded and conducted in a private space and at a time that will be convenient for participants. The questionnaire administration should take 40 min, while FGD and SSI sessions should take ~1 h.

### Analyses

#### Quantitative Analyses

As shown in Table 4, percentage change in knowledge will be calculated as percentage knowledge score at end line minus percentage knowledge score at baseline. Change in percentage of people screened will be calculated as percentage of population sample surveyed ever screened within the past 2 years at end line compared to percentage of population sample surveyed ever screened within the past 2 years at baseline. Percentage change graphs will also be plotted to illustrate the changes over time. We will use a two-sample *t*-test to compare the mean scores for baseline and end line. The analysis will also include summaries of categorical variables, which include response frequencies for each questionnaire item. An appropriate cut-off for knowledge will be chosen so that characteristics of people who meet the threshold can be compared to those who do not, both at baseline and end line testing. Proportion of community members who accepted screening will be calculated as the total number of people who were screened divided by the total number of people who met the threshold. Linkage to care for diabetes and hypertension will be calculated as the total number of persons treated for diabetes and hypertension divided by the total number of persons referred after screening positive for diabetes and hypertension.

Percentage of persons screened through each of the projects screening strategies who were linked to care will be calculated as the number of persons treated after referral from particular screening strategy divided by the total number of people treated after referral from screenings.

Percentage of patients with controlled blood pressure will be calculated at end line for care levels 2, 3, and 4 as the proportion of patients >60 years with blood pressure (BP) below 150/90 and patients <60 years with BP below 140/90. The study will also report the percentage of patients with a drop in hemoglobin A1c at end line for levels 2, 3, and 4. Retention rates for treatment of diabetes and hypertension will be calculated as number of patients seen 90 days from their last expected clinic visit divided by the total number of patients in care. Drug affordability will be measured, at baseline and end line, as the percentage of patients able to buy a whole month supply of prescribed select medicines.

#### Qualitative Analyses

The audio recordings will be transcribed and consequently coded using NVivo or a similar software. Prior to analysis, a research team member will listen to random sections of the recordings and compare them to the transcripts to verify accurate transcription. Then, three coders will look at the research questions, field notes and transcripts to familiarize themselves with the data. They will be guided by the research questions to develop a codebook through segmentation of thoughts found in the raw text. Each segment will be labeled with a descriptive code and definition that captures the ideas therein. The descriptive code labels will be assessed and similar patterns will be grouped together and labeled thematically. Analytic memos will track decision-making among coders as the codebook and themes are refined through subsequent stages of inductive development of themes. Quotes from transcripts will be used to provide vivid illustrations of the findings.

## Ethics and Dissemination

This study was reviewed and approved by the Moi University College of Health Sciences and Moi Teaching and Referral Hospital institutional Ethics Committee. It was also approved by the National Commission for Science and Technology (NACOSTI) and endorsed by relevant County and health facility leadership. The objectives of the research will be clearly explained to potential participants. Participation will be voluntary and informed consent will be sought from each participant. Those who choose not to participate in the study will have their decision respected. Participants will also be allowed to withdraw from the study at any point, without any consequences. In order to protect participant privacy, the research team will not use any identifying information when managing data, running data analysis or writing the study reports. All data will be kept in lockable cabinets and passwords will be used for data saved on computers. Participants will be given the contact details of the study's principal investigator so that they can get more clarification on the evaluation—as necessary.

The outcomes of the study will be made available to diverse stakeholders in Kenya, beginning with the communities of research and decision makers in Busia and Trans Nzoia. Specific dissemination for the communities and decision makers at the national, County and Sub-County levels will be carried out through mini workshops, community meetings, and government meetings. Findings and lessons learned will be provided and shared in various forms including written full reports, power point decks of cards, and policy briefs. In addition, study findings will be made available to the community of science through conferences and journal publications.

## Discussion

Similar to other low and medium income countries (LMICs), Kenya is experiencing the burden of NCD. The aim of the PIC4C model was to identify people with the four conditions and ensure their timely referral to, treatment and management at the appropriate service level (Health Center, Dispensary, Sub-County or County hospital). In this paper, we have described the protocol and appendices being used to conduct a process evaluation of the PIC4C model. The Ministry of Health, AMPATH NCD clinics, and PIC4C research team have worked collaboratively to ensure the pilot model succeeds. The process evaluation is directly built upon the expectations of the PIC4C model and will provide valuable experiences and perspectives from recipient communities. The use of two waves of data collection allow for a better picture of the model for it reduces possibilities of biases or misleading information.

This process evaluation has strengths and weaknesses. First, there are important lessons to be shared from the PIC4C process evaluation that are valuable to communities of research, local leadership, and broader academia. The PIC4C process evaluation protocol, methods, tools and study populations that were selected reflect what we find in other Kenyan counties and other similar LMICs. We therefore anticipate that this protocol is relevant to other settings. Secondly, the process evaluation is helping us illuminate aspects of the PIC4C Model that have worked well. There were also challenges and limitations that we have been experiencing. For instance, it would have been better to have a three-level monitoring and evaluation process: baseline, midterm, and post-intervention. This would have provided a better portrayal of inputs, all community and health facility activities, and associated outputs. However, due to the COVID-19 pandemic, the mid-term wave was not feasible. We therefore missed some opportunities during the implementation phase to observe and use resultant findings to inform users and the PIC4C team. Nonetheless, this process evaluation has been providing data and evidence that is useful for the current PIC4C pilot, and future anticipated scale ups of integrated care for NCDs in Kenya and beyond.

## Ethics Statement

The studies involving human participants were reviewed and approved by Moi University College of Health Sciences and Moi Teaching and Referral Hospital institutional Ethics Committee, Eldoret. The patients/participants provided their written informed consent to participate in this study.

## Author Contributions

VN, JK, and KT contributed to the conceptualization of the project and PIC4C process evaluation. VN, JK, HK, KT, GG, and OL contributed to the development of data collection materials, recruitment of participants, and data collection. VN, HK, and BM drafted the manuscript, and all authors edited and approved the final draft.

## Funding

This project was supported by the World Bank under the terms of grant number World Bank TFA5636 and Access Accelerated Initiative.

## Author Disclaimer

Contents are the authors' sole responsibility and do not necessarily represent interpretations by national, County, or funding stakeholders.

## Conflict of Interest

The authors declare that the research was conducted in the absence of any commercial or financial relationships that could be construed as a potential conflict of interest.

## Publisher's Note

All claims expressed in this article are solely those of the authors and do not necessarily represent those of their affiliated organizations, or those of the publisher, the editors and the reviewers. Any product that may be evaluated in this article, or claim that may be made by its manufacturer, is not guaranteed or endorsed by the publisher.
