# Supplementary material for: A Protocol on Using the RE-AIM Framework in the Process Evaluation of the Primary Health Integrated Care Project for Four Chronic Conditions in Kenya
Source: Front Public Health. 2022 Jan 12;9:781377. doi: 10.3389/fpubh.2021.781377 (PMC8790115; doi:10.3389/fpubh.2021.781377)
Supplement: Supplementary file 1 [file Data_Sheet_1.docx]

**SUPPLEMENTARY MATERIAL – TOOLS FOR THE PIC4C MODEL, KENYA**

**Supplementary File 1.** **Health Facility Questionnaire**

| **PIC4C STUDY Facility Questionnaire** |
| --- |
| **Questionnaire**  STUDY ID:  _______              Today’s Date: ___/___ /_____  (dd/mm/yyyy) |
|  |
| **Enrollment location:**  ☐Trans Nzoia              ☐  Busia  ☐Link facility name: _____________________  ☐ Level of care: ______________________ |
|  |
| **Respondent: Facility Manager** |
| 1. **Facility Information:**   How many providers of each of the following types work at this facility?   \| **Cadre** \| **Total** \| \| --- \| --- \| \| Consultant Physician/Internist \|  \| \| Medical officer/General doctor (Doctor without a specialization) \|  \| \| Clinical officer \|  \| \| Pharmacist \|  \| \| Kenya Registered nurse (KRN) \|  \| \| Registered midwife \|  \| \| Enrolled/community nurse \|  \| \| Nurse  attendant \|  \| \| Nutritionist Pharm-tech, lab tech, Records Other (specify) ______ \|  \| |
|  |
| Does your facility offer the following services?   \| SERVICE \| Diabetes \| Hypertension \| Cervical Cancer \| Breast Cancer \| \| --- \| --- \| --- \| --- \| --- \| \| Awareness \| ☐ \| ☐ \| ☐ \| ☐ \| \| Screening \| ☐ \| ☐ \| ☐ \| ☐ \| \| Treatment \| ☐ \| ☐ \| ☐ \| ☐ \| \| Others \| ☐ \| ☐ \| ☐ \| ☐ \| |
|  |
| 1. **Availability of equipment and supplies**   B1. Does the facility have ________today?  If so, is it working?   \| **Item** \| **Available?**  **(yes – 1,**  **no – 2)** \| **Number?**  **(yes – 1, no  2)** \| **Working?**  **(yes – 1, no  2)Don’t know…999** \| \| --- \| --- \| --- \| --- \| \| 1. Stethoscope \|  \|  \|  \| \| 2. Manual Blood pressure machine \|  \|  \|  \| \| 3. Automatic Blood pressure machine \|  \|  \|  \| \| 4. Thermometer \|  \|  \|  \| \| 5. Glucometer \|  \|  \|  \| \| 6. Glucose strips \|  \|  \|  \| \| 7. HbA1c machine \|  \|  \|  \| \| 8.Laboratory biochemistry machine \|  \|  \|  \| \| 9. Height meter \|  \|  \|  \| \| 10. Adult weighing scale \|  \|  \|  \| \| 11. Measuring tape \|  \|  \|  \| \| 12. ECG \|  \|  \|  \| \| 13. Ultrasound machine \|  \|  \|  \| \| 14.Speculums \|  \|  \|  \| \| 15. Leep equipment \|  \|  \|  \| \| 16. Gynecological bed \|  \|  \|  \| \| 17. Cryotherapy equipment \|  \|  \|  \| \| 18. Biopsy needles \|  \|  \|  \| \| 19. Sterilizer/autoclave \|  \|  \|  \| \| 20 Refrigerator \|  \|  \|  \| \| 21. X-ray machine \|  \|  \|  \| \| 22. Centrifuge \|  \|  \|  \|   B2. Does this facility have electricity? ☐ Yes ☐ No  If yes, is it working? ☐ Yes ☐ No  B3. Does this facility have piped running water? ☐ Yes ☐ No  If yes, is the water running today?☐ Yes ☐ No |
|  |
| **C. Data from medical records**  **Transition:** The next questions require me to speak to a records officer or someone who manages medical records for screening and treatment of hypertension, diabetes, breast and cervical cancer. The questions aim to determine; the estimates of your clients diagnosed with these conditions, patient data capture and how reporting is done in this facility.  **Enumerator:** Ask to speak to the staff member who manages medical records for hypertension, diabetes, cervical and breast cancer screening and treatment. Have him/her obtain information for questions C1-C3 from the medical records. Make sure to read out the consent statement to the records officer and obtain his/her consent prior to conducting the interview.  **Enumerator:** Ask to go through the medical records with the facility manager to obtain this information.  **Are there any education activities done on these conditions in past 6 months;**   \|  \| Yes \| No \| Number of activities \| No record of no. \| \| --- \| --- \| --- \| --- \| --- \| \| Diabetes \| ☐ \| ☐ \|  \|  \| \| Hypertension \| ☐ \| ☐ \|  \|  \| \| Breast Cancer \| ☐ \| ☐ \|  \|  \| \| Cervical Cancer \| ☐ \| ☐ \|  \|  \|   **Are there any screening activities for;**   \|  \| Yes \| No \| Number of activities \| No record of no. \| \| --- \| --- \| --- \| --- \| --- \| \| Diabetes \| ☐ \| ☐ \|  \|  \| \| Hypertension \| ☐ \| ☐ \|  \|  \| \| Breast Cancer \| ☐ \| ☐ \|  \|  \| \| Cervical Cancer \| ☐ \| ☐ \|  \|  \|   **In the past 3 months, were any patients seen at this health facility for these conditions?**  **If yes, how many?**   \|  \| Yes \| No \| Number of activities \| No record of no. \| \| --- \| --- \| --- \| --- \| --- \| \| Diabetes \| ☐ \| ☐ \|  \|  \| \| Hypertension \| ☐ \| ☐ \|  \|  \| \| Breast Cancer \| ☐ \| ☐ \|  \|  \| \| Cervical Cancer \| ☐ \| ☐ \|  \|  \|   **In the past 3 months, were any patients newly diagnosed for these conditions?**  **If yes, how many?**   \|  \| Yes \| No \| Number/Date \| No records \| \| --- \| --- \| --- \| --- \| --- \| \| Diabetes \| ☐ \| ☐ \|  \|  \| \| Hypertension \| ☐ \| ☐ \|  \|  \| \| Breast Cancer \| ☐ \| ☐ \|  \|  \| \| Cervical Cancer \| ☐ \| ☐ \|  \|  \|   **Does this facility collect data on these conditions in a specific register?**  **If Yes, when was the last time it was updated?**   \|  \| Yes \| No \| Number/Date \| No records \| \| --- \| --- \| --- \| --- \| --- \| \| Diabetes \| ☐ \| ☐ \|  \|  \| \| Hypertension \| ☐ \| ☐ \|  \|  \| \| Breast Cancer \| ☐ \| ☐ \|  \|  \| \| Cervical Cancer \| ☐ \| ☐ \|  \|  \|   **Does this facility report indicators on these conditions in the MOH 705b, and/or 717**  **If yes, when was the last time any of them was filled?**   \|  \| Yes \| No \| Date \| No records \| \| --- \| --- \| --- \| --- \| --- \| \| Diabetes \| ☐ \| ☐ \|  \|  \| \| Hypertension \| ☐ \| ☐ \|  \|  \| \| Breast Cancer \| ☐ \| ☐ \|  \|  \| \| Cervical Cancer \| ☐ \| ☐ \|  \|  \|   **In this facility, clinical encounter records for patients with these conditions are captured in which of the following formats?**   1. Patient books B. Patient files C. Electronic Medical records D. Pieces of paper   E. Others   \|  \| A \| B \| C \| D \| E \| No clinical records \| \| --- \| --- \| --- \| --- \| --- \| --- \| --- \| \| Diabetes \| ☐ \| ☐ \| ☐ \| ☐ \| ☐ \| ☐ \| \| Hypertension \| ☐ \| ☐ \| ☐ \| ☐ \| ☐ \| ☐ \| \| Breast Cancer \| ☐ \| ☐ \| ☐ \| ☐ \| ☐ \| ☐ \| \| Cervical Cancer \| ☐ \| ☐ \| ☐ \| ☐ \| ☐ \| ☐ \| |
|  |
| 1. **Availability of medication**   Enumerator: Ask to go to the dispensary/pharmacy or place where drugs are kept within the facility. Make sure you see the medications before marking them down.  CHECK WHETHER THE FOLLOWING DRUGS ARE IN STOCK. FOR EACH DRUG, ATTEMPT TO DIRECTLY OBSERVE WHETHER IT IS IN STOCK. IF THIS IS NOT POSSIBLE, ASK WHETHER IT IS IN STOCK. IF IT IS NOT IN STOCK, ASK WHETHER THEY PROVIDE THE DRUG BUT ARE OUT OF STOCK*  **Availability**: 1-In stock (observed) 2-In stock (reported) 3-Not available now but usually available 4-Not stocked   \| Drug \| Availability \| Drug \| Availability \| \| --- \| --- \| --- \| --- \| \| 1.   Hydrochlorothiazide \|  \| 9. Aspirin \|  \| \| 2.   Amlodipine \|  \| 10. Insulin \|  \| \| 3.   Nifedipine \|  \| 11. Metformin \|  \| \| 4.   Propranolol \|  \| 12. Glibenclamide \|  \| \| 5.   Enalapril \|  \| 13. Amitrypline \|  \| \| 6.   Methyldopa \|  \| 14. Statin \|  \| \| 7.   Atenolol \|  \| 15. Lasix \|  \| \| 8.   Losartan \|  \| 16. Glimepiride \|  \| |
| **Transition**: For the next questions, I’d like to speak with the health providers who mostly run the clinics. The questions that I will ask will assess the providers’ knowledge about hypertension, diabetes, breast and cervical cancer. The questions are not intended to embarrass the provider, but the findings will help determine if there is a need to strengthen knowledge on these diseases among providers.  May I speak to the person who mostly attends to hypertensive clients if different from you? |
|  |
| **Provider consent if different from the facility manager**  **Respondent**: Provider most likely to attend to patients with diabetes, hypertension breast or cervical cancer.  What is the highest level of medical training you have attained?  ☐Consultant Physician/Internist  ☐Medical officer/General doctor (Doctor without a specialization)  ☐Clinical officer  ☐Bachelor Science nurse  ☐Kenya Registered nurse (KRN)  ☐Registered midwife  ☐Enrolled/community nurse  ☐Nurse attendant  ☐Other (specify) |
|  |
| **Provider knowledge**  What are the symptoms and signsof high blood pressure?  (Circle all mentioned. PROBE IN DETAIL. DO NOT READ POSSIBLE RESPONSES)   \| ☐ Severe headache \| ☐ Fatigue or confusion \| \| --- \| --- \| \| ☐ Vision problems \| ☐ Chest pain \| \| ☐ Difficulty breathing \| ☐ Irregular heartbeat \| \| ☐ Blood in the urine \| ☐ Pounding in your chest, neck, or ears \| \| ☐ Numbness \| ☐ Frequent urination \| \| ☐ Snoring \| ☐ Swelling of legs \| \| ☐ Excessive sweating \| ☐ Nose bleeding \| \| ☐ Dizziness \| ☐ Excessive thirst \| \| ☐ Breathing fast \| ☐ Palpitation \| \| ☐ Anxiety/bad temper \| ☐ No symptoms \| \| ☐ Other (Specify) \|  \| |
| Approximately what percentage of people with hypertension display no symptoms of hypertension?  ☐ 0% ☐ < 50% ☐ >50% |
| What are the risk factors for hypertension? (Multiple Response. PROBE IN DETAIL. DO NOT READ. )   \| ☐ Age \| ☐ Family history \| \| --- \| --- \| \| ☐ High BMI/Obesity/Overweight \| ☐ Lack of physical activity \| \| ☐ Use of tobacco products \| ☐ High salt intake \| \| ☐ Low potassium intake \| ☐ Low vitamin D intake \| \| ☐ High alcohol consumption \| ☐ High stress \| \| ☐ Use of contraceptives \| ☐ Pregnancy \| \| ☐ Eating  raw (uncooked)/ salt \| ☐ Processed salt \| \| ☐ Diet low in fruits  and vegetables \| ☐ No risk factors \| \| ☐ Other (specify) :___________ \|  \| |
| What are some of the ways that high blood pressure can be controlled? (Multiple Response Do not prompt)   \| ☐ Reduce salt \| ☐ Reduce alcohol \| \| --- \| --- \| \| ☐ Reduce smoking \| ☐ Weight loss \| \| ☐ Reduce sugar \| ☐ Medication \| \| ☐ Exercise \| ☐ Reduce the amount of red meat eaten \| \| ☐ Stop use of chemical contraceptives \| ☐ Reduce stress levels \| \| ☐ Avoid junk foods \| ☐ Avoid eating fatty foods \| \| ☐ Use of liquid oil for cooking \| ☐ Drinking lots of water \| \| ☐ Eat diets rich in fruits and vegetables \| ☐ Other (specify) ___________ \| |
| E5. What are the potential medically known consequences of hypertension? (Multiple Response. Do not prompt)   \| ☐ Heart attack \| ☐ Stroke \| \| --- \| --- \| \| ☐ Aneurysm \| ☐ Heart failure \| \| ☐ Trouble with memory \| ☐ Local myth \| \| ☐ Local myth \| ☐ Erectile dysfunction \| \| ☐ Death \| ☐ Complications during delivery for pregnant women \| \| ☐ Chronic fatigue \| ☐ Loss of sight \| \| ☐ Increased risk of miscarriage \| ☐ Renal Disease \| \| ☐ Nothing \| ☐ Other (specify) ______________ \| |
| What is the systolic blood pressure threshold for *pre-hypertension*?  That is, if a patient’s systolic blood pressure is above this value, the person should be diagnosed as having prehypertension.  ☐ Below 120☐ 120 – 139☐ Don’t know |
| What is the systolic blood pressure threshold for *hypertension*?  That is, if a patient’s systolic blood pressure is above this value, the person should be diagnosed as having hypertension?  ☐Below 140☐140+☐Don’t know |
| What is the diastolic blood pressure threshold for *pre-hypertension*?  That is, if a patient’s diastolic blood pressure is above this value, the person should be diagnosed having prehypertension?  ☐Below 80☐ 80 -89☐ Don’t know |
| What is the diastolic blood pressure threshold for *hypertension*?  That is, if a patient’s diastolic blood pressure is above this value, the person should be diagnosed as having hypertension?  ☐Below 90☐90+ ☐Don’t know |
| What is the systolic blood pressure threshold for severe hypertension?  That is, if a patient’s systolic blood pressure is above this value, the person should be immediately referred to a hospital.  ☐ Below 180 ☐ 180+ ☐ Don’t know |
| What is the diastolic blood pressure threshold for severe hypertension?  That is, if a patient’s diastolic blood pressure is above this value, the person should be immediately referred to a hospital.  ☐ Below 110☐ 110 and above☐ Don’t know |
| Which of the following statements best describes how you interpret blood pressure readings when diagnosing a patient as hypertensive  ☐ Both systolic and diastolic readings should be higher than the recommended threshold  ☐ At least one readings should be higher than the recommended threshold |
| What procedure do you follow to diagnose hypertension in a patient? (Multiple Spontaneous Response- Do not read!)  ☐ Take their blood pressure reading once  ☐ Take their blood pressure reading more than once during the same visit  ☐ Take their blood pressure on multiple occasions  ☐ Other (Specify)  ☐ Don’t know |
| What is the minimum amount of time that should elapse between blood pressure readings during the same visit?  [_____] Minutes☐ Don’t know |
|  |
| **SECTION 2 DIABETES –KNOWLEDGE** |
| What are the signs and symptoms of diabetes? Circle all mentioned   \| ☐ Excessive thirst \| ☐ Frequent Urination \| \| --- \| --- \| \| ☐ Frequent hunger \| ☐ Vision Problems \| \| ☐ Wounds that take time to heal \| ☐ Confusion \| \| ☐ Coma \| ☐ Tingling sensation \| \| ☐ Excessive urination at night \| ☐ Fatigue \| \| ☐ Skin infections \| ☐ Unexplained Weight loss \| \| ☐ Recurrent candida infection \| ☐ Dental caries \| \| ☐ No symptoms \| ☐ Don’t know \| |
| Approximately what percentage of people with diabetes show no symptom of diabetes?  ☐ 0 %☐ <50%☐ >50% |
| What are the risk factors for diabetes?   \| ☐ Age \| ☐ Family history \| \| --- \| --- \| \| ☐ High BMI/Obesity/Overweight \| ☐ Lack of physical activity \| \| ☐ Use of tobacco products \| ☐ High alcohol consumption \| \| ☐ High stress \| ☐ Use of contraceptives \| \| ☐ Pregnancy \| ☐ No risk factors \| \| ☐ Diet low in fruits  and vegetables \| ☐ Use of certain drugs \| \| ☐ Ethnicity \| ☐ Other hormone related diseases \| \| ☐ Hypertension \| ☐ Other (specify) ___________ \| \| ☐ Don’t know \|  \| |
| How do you diagnose pre diabetes?   \| ☐ FBS Below 6.1 mmol/L \| ☐ FBS  6.1-6.9mmol/l \| \| --- \| --- \| \| ☐ FBS > 6.9 \| ☐ OGTT < 7.8 \| \| ☐ OGTT 7.8- 11.1 \| ☐ OGTT > 11.1 \| \| ☐ HbA1c < 5.6% \| ☐ HbA1c 5.6-6.5% \| \| ☐ HbA1c > 6.5% \| ☐ Don’t know \| |
| How do you diagnose diabetes   \| ☐Symptoms of diabetes with RBS> 11.1mmol/l \| ☐ RBS >11.1mmol/l \| \| --- \| --- \| \| ☐ RBS < 11.1 \| ☐ OGTT > 11.1 mmol/l \| \| ☐ OGTT < 11.1 mmol/l \| ☐ FBS >7.0mmol/l \| \| ☐ FBS < 7.0 mmol/l \| ☐ HbA1c> 6.5% \| \| ☐ HbA1c < 6.5% \| ☐ Don’t know \| |
| What are some of the ways that diabetes can be controlled? (Multiple Response Do not prompt)   \| ☐ Reduce salt \| ☐ Reduce alcohol \| \| --- \| --- \| \| ☐ Reduce smoking \| ☐ Weight loss \| \| ☐ Reduce sugar \| ☐ Medication \| \| ☐ Exercise \| ☐ Reduce the amount of red meat eaten \| \| ☐ Stop use of chemical contraceptives \| ☐ Reduce stress levels \| \| ☐ Avoid junk foods \| ☐ Avoid eating fatty foods \| \| ☐ Use of traditionally prepared African salts \| ☐ Use of liquid oil for cooking \| \| ☐ Drinking lots of water \| ☐ Diets rich in fruits \| \| and vegetables \| ☐ Other (specify) _________ \| \| ☐ Don’t know \|  \| |
| What are the potential medically known consequences of diabetes? (Multiple Response. Do not prompt)   \| ☐ Heart attack \| ☐ Stroke \| \| --- \| --- \| \| ☐ Aneurysm \| ☐ Heart failure \| \| ☐ Trouble with memory \| ☐ Neuropathy \| \| ☐ Local myth 1 \| ☐ Local myth 2 \| \| ☐ Erectile dysfunction \| ☐ Death \| \| ☐Complications during delivery for pregnant women \| ☐ Chronic fatigue \| \| ☐ Loss of sight \| ☐ Increased risk of miscarriage \| \| ☐ Renal Disease \| ☐ Amputations \| \| ☐ No complication \| ☐ Other (specify) ____________ \| \| ☐ Don’t know \|  \| |
|  |
| **SECTION 3: BREAST CANCER – KNOWLEDGE** |
| What are the risk factors for breast cancer   \| ☐ Being female \| ☐ Radiation exposure \| \| --- \| --- \| \| ☐ Having first child at an older age \| ☐ Having never been pregnant \| \| ☐ Drinking alcohol \| ☐ Overweight/obese \| \| ☐ Family planning pills \| ☐ Smoking/Tobacco use \| \| ☐ Alcohol use \| ☐ Inherited risk \| \| ☐ Increasing Age \| ☐ Failure to breast feed \| \| ☐ hormone replacement therapy \| ☐ Other  _______________ \| \| ☐ Do not know answer/No answer \|  \| |
| What are the signs and symptoms of breast cancer?   \| ☐ Discharge from the nipple other than breast milk \| ☐ Breast lump \| \| --- \| --- \| \| ☐ Nipple tenderness \| ☐ Change in breast skin texture \| \| ☐ Change in shape/size \| ☐ Breast pain \| \| ☐ Swelling of breast \| ☐ Inverted nipple \| \| ☐ Skin irritation or dimpling \| ☐ Darkening, scalyness, thickening of breast skin nipple \| \| ☐ Others \| ☐ I don’t know \| |
| How do you screen for breast cancer?   \| ☐ Self Breast Examination \| ☐ Clinical Breast Examination \| \| --- \| --- \| \| ☐ Ultra sound \| ☐ Mammogram \| \| ☐ MRI \| ☐ FNA \| \| ☐ Biopsy \|  \| |
| How is breast cancer diagnosed?  ☐ Histology  ☐ Cytology |
| Describe breast cancer treatment?   \| ☐ Mastectomy alone \| ☐ Mastectomy plus chemotherapy \| \| --- \| --- \| \| ☐ Mastectomy plus radiotherapy \| ☐ Chemotherapy only \| \| ☐ Radiotherapy only \| ☐ Mastectomy, Radio and chemotherapy combined \| |
|  |
| **SECTION 4. CERVICAL CANCER KNOWLEDGE** |
| What are the risk factors for cervical cancer   \| ☐ HPV infection \| ☐ Having many sexual partners \| \| --- \| --- \| \| ☐ Physical inactivity \| ☐ Excessive drinking of alcohol \| \| ☐ Overweight/obese \| ☐ long term use of birth control pills \| \| ☐ Smoking/Tobacco use \| ☐ Engaging in early sexual contact \| \| ☐ Inherited risk \| ☐ HIV or other immunosuppressive diseases infection \| \| ☐ Multi gravidy \| ☐ Herpes \| \| ☐ Other  _______________ \| ☐ Do not know answer/No answer \| |
| What are the signs and symptoms of cervical cancer?   \| ☐ Blood spots/light bleeding between periods \| ☐ Menstrual periods heavier and longer than usual \| \| --- \| --- \| \| ☐ Bleeding after intercourse \| ☐ Fatigue \| \| ☐ Pain during sexual intercourse \| ☐ Bleeding after menopause \| \| ☐ Increased vaginal discharge \| ☐ Pelvic pain \| \| ☐ I don’t know \| ☐ Others \| |
| How do you screen for cervical cancer   \| ☐ Speculum observation \| ☐ VIA \| \| --- \| --- \| \| ☐ VILI \| ☐ Pap smear \| \| ☐ HPV blood test \| ☐ Urinalysis \| \| ☐ VE \|  \| |
| How is cervical cancer diagnosed  ☐ Colposcopy  ☐ Biopsy  and histology  ☐ Inspection  ☐ Pap smear and cytology  ☐ Don’t know |
| Describe cervical cancer treatment  ☐ LEEP  ☐ Cryotherapy  ☐ Hysterectomy alone..........................  ☐ Hysterectomy  plus chemotherapy  ☐ Hysterectomy plus radiotherapy  ☐ Chemotherapy only  ☐ Radiotherapy only  ☐ Radio and chemotherapy combined |
|  |
| Attitudes and Practices |
| F1. Are there certain days/clinics when you provide care specifically for patients with -----  Diabetes                  ☐ Yes    ☐ No  Hypertension    ☐ Yes    ☐ No  Breast Cancer              ☐ Yes   ☐ No  Cervical Cancer            ☐ Yes    ☐ No  ***(If no skip to F3)*** |
| What activities does this facility conduct to make people aware of the Hypertension clinic? (circle all mentioned)  ☐ Outreach  ☐ Advertise through barazas  ☐ Advertise through church  ☐ Client education/counseling during facility visits  ☐ Education during health days  ☐ Other(specify) |
| What activities does this facility conduct to make people aware of the diabetes clinic? (circle all mentioned)  ☐ Outreach  ☐ Advertise through barazas  ☐ Advertise through church  ☐ Client education/counseling during facility visits  ☐ Education during health days  ☐ Other(specify)……………… |
| What activities does this facility conduct to make people aware of cervical cancer clinic? (circle all mentioned)  ☐ Outreach through CHVs  ☐ Advertise through barazas  ☐ Advertise through church  ☐ Client education/counseling during facility visits  ☐ Education during health days  ☐ Outreach to chamas  ☐ Education in MCH  ☐ Other(specify)…………… |
| F3.When visiting a health facility for any condition, how often is a patient’s blood pressure taken?  ☐ Every visit ☐ Less than every visit |
|  |
| Please indicate how strongly you agree with this statement (strongly agree, agree, somewhat agree, disagree):   \|  \| Condition \| strongly agree \| agree \| somewhat agree \| disagree \| \| --- \| --- \| --- \| --- \| --- \| --- \| \| Awareness of (mention condition)is low among patients that come to this facility \| Diabetes \| ☐ \| ☐ \| ☐ \| ☐ \| \| Hypertension \| ☐ \| ☐ \| ☐ \| ☐ \| \| Breast Cancer \| ☐ \| ☐ \| ☐ \| ☐ \| \| Cervical Cancer \| ☐ \| ☐ \| ☐ \| ☐ \| \| Educating patients on the importance of adherence to medication for (mention condition) is difficult because they only take pills until they feel better \| Diabetes \| ☐ \| ☐ \| ☐ \| ☐ \| \| Hypertension \| ☐ \| ☐ \| ☐ \| ☐ \| \| Breast Cancer \| ☐ \| ☐ \| ☐ \| ☐ \| \| Cervical Cancer \| ☐ \| ☐ \| ☐ \| ☐ \| \| If  patients with (mention condition)understood the risks, they would adhere to their medication \| Diabetes \| ☐ \| ☐ \| ☐ \| ☐ \| \| Hypertension \| ☐ \| ☐ \| ☐ \| ☐ \| \| Breast Cancer \| ☐ \| ☐ \| ☐ \| ☐ \| \| Cervical Cancer \| ☐ \| ☐ \| ☐ \| ☐ \| \| (mention condition)is preventable \| Diabetes \| ☐ \| ☐ \| ☐ \| ☐ \| \| Hypertension \| ☐ \| ☐ \| ☐ \| ☐ \| \| Breast Cancer \| ☐ \| ☐ \| ☐ \| ☐ \| \| Cervical Cancer \| ☐ \| ☐ \| ☐ \| ☐ \| \| God or a higher power ultimately determines one gets (mention condition) \| Diabetes \| ☐ \| ☐ \| ☐ \| ☐ \| \| Hypertension \| ☐ \| ☐ \| ☐ \| ☐ \| \| Breast Cancer \| ☐ \| ☐ \| ☐ \| ☐ \| \| Cervical Cancer \| ☐ \| ☐ \| ☐ \| ☐ \| \| Patients with (mention condition)are tiring \| Diabetes \| ☐ \| ☐ \| ☐ \| ☐ \| \| Hypertension \| ☐ \| ☐ \| ☐ \| ☐ \| \| Breast Cancer \| ☐ \| ☐ \| ☐ \| ☐ \| \| Cervical Cancer \| ☐ \| ☐ \| ☐ \| ☐ \| \| (mention condition)affect people who do not take care of their health \| Diabetes \| ☐ \| ☐ \| ☐ \| ☐ \| \| Hypertension \| ☐ \| ☐ \| ☐ \| ☐ \| \| Breast Cancer \| ☐ \| ☐ \| ☐ \| ☐ \| \| Cervical Cancer \| ☐ \| ☐ \| ☐ \| ☐ \| \| Patients with(mention condition) are unteachable \| Diabetes \|  \|  \|  \|  \| \| Hypertension \| ☐ \| ☐ \| ☐ \| ☐ \| \| Breast Cancer \| ☐ \| ☐ \| ☐ \| ☐ \| \| Cervical Cancer \| ☐ \| ☐ \| ☐ \| ☐ \| \| When people with (mention condition)understand their condition, they can adhere to treatment \| Diabetes \| ☐ \| ☐ \| ☐ \| ☐ \| \| Hypertension \| ☐ \| ☐ \| ☐ \| ☐ \| \| Breast Cancer \| ☐ \| ☐ \| ☐ \| ☐ \| \| Cervical Cancer \| ☐ \| ☐ \| ☐ \| ☐ \| \| If supported,people with (mention condition) can acquire self-management skills. \| Diabetes \| ☐ \| ☐ \| ☐ \| ☐ \| \| Hypertension \| ☐ \| ☐ \| ☐ \| ☐ \| \| Breast Cancer \| ☐ \| ☐ \| ☐ \| ☐ \| \| Cervical Cancer \| ☐ \| ☐ \| ☐ \| ☐ \| \| The resources in our current health system are too few to do anything (mention condition) \| Diabetes \| ☐ \| ☐ \| ☐ \| ☐ \| \| Hypertension \| ☐ \| ☐ \| ☐ \| ☐ \| \| Breast Cancer \| ☐ \| ☐ \| ☐ \| ☐ \| \| Cervical Cancer \| ☐ \| ☐ \| ☐ \| ☐ \| \| (mention condition) cannot be managed at this level of healthcare \| Diabetes \| ☐ \| ☐ \| ☐ \| ☐ \| \| Hypertension \| ☐ \| ☐ \| ☐ \| ☐ \| \| Breast Cancer \| ☐ \| ☐ \| ☐ \| ☐ \| \| Cervical Cancer \| ☐ \| ☐ \| ☐ \| ☐ \| \| I am confident in my ability to manage (mention condition) \| Diabetes \| ☐ \| ☐ \| ☐ \| ☐ \| \| Hypertension \| ☐ \| ☐ \| ☐ \| ☐ \| \| Breast Cancer \| ☐ \| ☐ \| ☐ \| ☐ \| \| Cervical Cancer \| ☐ \| ☐ \| ☐ \| ☐ \| \| I am confident that my colleagues in this facility can manage (mention condition) \| Diabetes \| ☐ \| ☐ \| ☐ \| ☐ \| \| Hypertension \| ☐ \| ☐ \| ☐ \| ☐ \| \| Breast Cancer \| ☐ \| ☐ \| ☐ \| ☐ \| \| Cervical Cancer \| ☐ \| ☐ \| ☐ \| ☐ \| |
|  |
| ***Enumerator: For the following two questions, you should use observational skills to answer the questions. Do not ask the provider these questions.*** |
| \|  \|  \| **Yes** \| **No** \| \| --- \| --- \| --- \| --- \| \| Surveyor instructions: please note if there are client-focused diabetes, hypertension, breast or cervical cancer posters on the wall of the facility, on the desk, or any other place in the facility \| Diabetes \| ☐ \| ☐ \| \| Hypertension \| ☐ \| ☐ \| \| Breast Cancer \| ☐ \| ☐ \| \| Cervical Cancer \| ☐ \| ☐ \| \| Surveyor instructions: please note if there are provider hypertension, diabetes, breast  and cervical cancer algorithm posters on the wall of the facility, on the desk, or any other place in the facility \| Diabetes \| ☐ \| ☐ \| \| Hypertension \| ☐ \| ☐ \| \| Breast Cancer \| ☐ \| ☐ \| \| Cervical Cancer \| ☐ \| ☐ \| |
|  |
| **Enumerator: Ask to be shown MOH data collection and reporting tools for diabetes, hypertension, breast cancer and cervical cancer. Complete the following table from your findings.**   \| Tool \| Availability \| \| Completion \| \| \| \| --- \| --- \| --- \| --- \| --- \| --- \| \| Yes \| No \| Fully filled \| Partially filled \| Not filled \| \| Screening Register \| ☐ \| ☐ \| ☐ \| ☐ \| ☐ \| \| Hypertension register \| ☐ \| ☐ \| ☐ \| ☐ \| ☐ \| \| Hypertension clinic appointment register \| ☐ \| ☐ \| ☐ \| ☐ \| ☐ \| \| Diabetes register \| ☐ \| ☐ \| ☐ \| ☐ \| ☐ \| \| Diabetes clinic appointment register \| ☐ \| ☐ \| ☐ \| ☐ \| ☐ \| \| Breast cancer register \| ☐ \| ☐ \| ☐ \| ☐ \| ☐ \| \| Breast Cancer clinic appointment register \| ☐ \| ☐ \| ☐ \| ☐ \| ☐ \| \| Cervical Cancer register \| ☐ \| ☐ \| ☐ \| ☐ \| ☐ \| \| Cervical cancer clinic appointment register \| ☐ \| ☐ \| ☐ \| ☐ \| ☐ \| |

**Supplementary File 2: Patient Reception and Vital Signs’ Assessment Checklist (Observation process mapping)**

**a. Reception**

Registration and records:

Check for the following

-Patient greeted by triage nurse, referral card or clinic card number used to retrieve patient's’ file or electronic records.

-For new patients, correct registration process done and clinic card issued.

-A queue number issued to allow organized queue management.

**b. Measurement taking**

Check that machines are functional and supplies are available:

[ ] BP machine

[ ] Glucometer

[ ] Strips

[ ] Prickers

[ ] Swabs (cotton wool and spirit)

[ ] Waste receivers

Explain and correctly perform blood pressure procedures:

[ ] Participant was sitting with feet on the floor

[ ] Participant’s arm was at heart level

[ ] Participant removed extra clothing (coat/jacket must be removed; long sleeve shirts may remain on)

[ ] Participant was encouraged to relax

[ ] Participant was not talking during measurement

[ ] If participant was talking, measurement was repeated

[ ] BP cuff was well placed above the elbow and admitting two fingers

[ ] Correct size of BP cuff was used

[ ] BP is repeated if elevated

Repeat reading was at least 5 minutes later

Measure participant height:

[ ] Participant was not wearing shoes

[ ] Participant was not wearing headgear, like a hat

[ ] Tape measure was correctly used

Measure participant weight:

[ ] Scale was placed on a firm, flat surface

[ ] Participant was not wearing shoes

[ ] Participant was not wearing heavy outer layers

Measure waist circumference:

[ ] Participant was standing with feet together and arms relaxed at sides

[ ] Measurement was taken at the midpoint of the last palpable rib and the top of the hip bone

Explain and correctly measure blood glucose using glucometer:

[ ] Asked if patient is fasting

[ ] Placed the glucose strip in the glucometer and hit run

[ ] Performed finger prick

[ ] Dropped blood drop onto glucostrip receiver point

[ ] Waited a few seconds to see the results

[ ] Disposal of the pricker, swabs and used strips correctly done.

[ ] Result recorded immediately

**c. Clinician observation**

**Note: Observe one physician-patient interaction. Choose a patient who is approximately in the middle of the queue.*

Clinician direct observation:

[ ] Clinician greeted the patient with welcoming words

[ ] Clinician referred to the BP and blood sugar measurements

[ ] Clinician asked patient about symptoms/how they have been doing

[ ] Clinician listened to patient without interrupting

[ ] Clinician asked about adherence to behavioral recommendations (diet, exercise, etc)

[ ] Patients referred for further assessment for cervical or breast cancer, clinician had all the necessary tools set out for procedure.

[ ] OR clinician explained why the procedure could not be done at that time and booked on another specific date or to another specific clinic.

[ ] Clinician explained the procedure to patient.

[ ] Clinician allowed patient time to ask questions, and tried to answer.

[ ] Clinician asked about medication adherence

[ ] Clinician adjusted medication

[ ] Clinician captured measurements and medication adjustment, either on paper or electronically through AMRS.

[ ] Clinician told patient their next appointment date

**d. Other clinical observations**

[ ] All of the medications needed for DM and HTN were dispensed

[ ] Necessary laboratory tests were done or appropriate referral done.

[ ] Time taken from one patient’s arrival to time they leave the clinic.

[ ] Time first patient is seen.

[ ] Time last patient is seen.

**Supplementary File 3: Education and Screening Observation Checklist**

**a. Group education sessions before screening**

Attendance:

[ ] Attendance was taken by the CHV or CHP

● Time session began: _____

● Time session ended: _____

Content:

[ ] CHV/CHP gave short introduction with welcoming words

[ ] CHV/CHP provided 10-15 minutes health education talk

[ ] Health education topic was appropriate

Group facilitation:

|  | Met Participant Needs? | | | | |
| --- | --- | --- | --- | --- | --- |
|  | 1 (no) | 2 | 3 (ok) | 4 | 5 (yes) |
| Did the facilitator allow sufficient discussion? |  |  |  |  |  |
| Did the facilitator encourage group participation? |  |  |  |  |  |
| Did the facilitator help bring out new group ideas? |  |  |  |  |  |
| Did the facilitator help close out discussions? |  |  |  |  |  |
| Would you accept this facilitator again? |  |  |  |  |  |

[ ] Participants seemed engaged throughout the discussion

[ ] CHV gave positive encouragement to group members

[ ] CHV directed any medical questions towards clinician

**Note: CHV should not give any clinical advice)*

**b. Screening sessions**

Measurement taking:

Check that machines are functional and supplies are available:

[ ] BP machine

[ ] Glucometer

[ ] Strips

[ ] Prickers

[ ] Swabs (cotton wool and spirit)

[ ] Waste receivers

Did CHV/CHP:

1. Explain and correctly perform blood pressure procedures:

[ ] Participant was sitting with feet on the floor

[ ] Participant’s arm was at heart level

[ ] Participant removed extra clothing (coat/jacket must be removed; long sleeve shirts may remain on)

[ ] Participant was encouraged to relax

[ ] Participant was not talking during measurement

[ ] If participant was talking, measurement was repeated

[ ] BP cuff was well placed above the elbow and admitting two fingers

[ ] Correct size of BP cuff was used

[ ] BP is repeated if elevated

[ ] Repeat reading was at least 5 minutes later

2. Measure Participant Height:

[ ] Participant was not wearing shoes

[ ] Participant was not wearing headgear, like a hat

[ ] Tape measure was correctly used

3. Measure Participant Weight:

[ ] Scale was placed on a firm, flat surface

[ ] Participant was not wearing shoes

[ ] Participant was not wearing heavy outer layers

4. Measure Waist Circumference:

[ ] Participant was standing with feet together and arms relaxed at sides

[ ] Measurement was taken at the midpoint of the last palpable rib and the top of the hip bone

5. Explain and correctly measure Blood Glucose using Glucometer:

[ ] Asked if patient is fasting

[ ] Placed the glucose strip in the glucometer and hit run

[ ] Performed finger prick

[ ] Dropped blood drop onto glucostrip receiver point

[ ] Waited a few seconds to see the results

[ ] Disposal of the pricker, swabs and used strips correctly done.

[ ] Result recorded immediately

**c. Referral process**

Did the CHV/CHP:

[ ] Explain normal values to patients correctly.

[ ] Refer patients with readings above stipulated cut-offs appropriately.

[ ] Provide referred patients with detailed referral cards with specific clinic, and clinic dates written.

[ ] Give patients time to ask questions and choose a preferred facility before dismissing them.

**Supplementary File 4: Written Test/Random Knowledge Test among Community health volunteers (CHVs), Community health promoters (CHPs) and Clinicians**

**Section A. CHVs/CHPs Written Test**

**Hypertension**

Choose the ONE best answer

1. What is high blood pressure?

a. When there is more pressure against the walls of blood vessels than we need

b. When there is too much blood inside the brain

c. Having too much fluid inside the body

d. When the heart is too big

2. What causes high blood pressure?

a. Drinking too much water

b. Eating too much salt

c. Exercising daily

d. Eating a lot of fruits.

e. Eating a lot of spicy foods.

3. Which of the following is usually NOT a symptom of high blood pressure?

a. Headache

b. Double vision

c. Chest pain

d. Shortness of breath

e. Fever

4. What is one strategy that a patient can use to manage her high blood pressure without medications?

a. Smoke cigarettes

b. Drink more alcohol

c. Reduce body weight

d. Sit down more

e. Avoid the company of other people

5. Which of the following is NOT a complication of untreated high blood pressure?

a. Stroke

b. Heart attack

c. Weight loss

d. Kidney problems

Circle TRUE or FALSE for questions 6 through 10

6. TRUE or FALSE: Peeling skin is a possibly serious side effect from high blood pressure medication.

7. TRUE or FALSE: It is more expensive for a patient to treat high blood pressure than manage complications from high blood pressure.

8. TRUE or FALSE: All patients with high blood pressure have symptoms that tell them when their blood pressure is high.

9. TRUE or FALSE: It is okay to get medications for high blood pressure from the chemist without a prescription from a healthcare provider.

10. TRUE or FALSE: Management of high blood pressure with medication is most effective when lifestyle modifications are also implemented

**Diabetes**

Choose the ONE best answer

1. What is diabetes?

a. When you eat too much sugar

b. When your body does not make or use insulin well

c. When you are overweight

d. When you eat too much salt

2. What causes diabetes?

a. Exercising daily

b. Eating a lot of fruits

c. Eating a lot of fats

d. Not drinking enough water

e. A combination of lifestyle and genetic factors

3. Which of the following is usually NOT a symptom of diabetes?

a. Increased urination

b. Vision changes

c. Weight loss

d. Diarrhea

e. Infections that take longer to heal

4. What is one strategy that a patient can use to manage her diabetes without medications?

a. Sit down more

b. Smoke cigarettes

c. Move more

d. Gain weight

e. Drink more alcohol

5. Which of the following is NOT a complication of untreated diabetes?

a. Eye damage leading to blindness

b. Numbness and pain in feet, legs, and arms

c. Difficulty hearing

d. Heart disease

e. Kidney disease

Circle TRUE or FALSE for questions 6 through 10

6. TRUE or FALSE: Prediabetes always turns into diabetes.

7. TRUE or FALSE: Hypertension is a risk factor for diabetes.

8. TRUE or FALSE: Blood sugar that stays high leads to long-term problems from diabetes.

9. TRUE or FALSE: Eating a diet with more starches such as bread and ugali helps to manage diabetes

10. TRUE or FALSE: Diabetic patients should take extra care of their feet by washing and looking at them.

**Breast cancer**

Choose the one best answer.

1. Who can get breast cancer?

a. Only people with a family history of breast cancer.

b. Both women and men

c. Women who have big breasts but not those with small breasts.

d. All people can get breast cancer

e. Both B and D.

2. What are the symptoms of breast cancer?

a. Breast lump

b. Nipple discharge.

c. Abnormal wrinkling or discoloration of skin over the breast.

d. Large breasts.

e. A, B and C.

3. How does one screen for breast cancer?

a. By going to hospital to do a CT scan

b. By doing self-breast examination frequently.

c. By doing a chest x ray.

d. By doing a blood test.

e. None of the above

4. Which of the following is true about breast lumps?

a. All breast lumps are due to cancer.

b. All large breast lumps are due to cancer.

c. Removing a breast lump causes cancer to spread.

d. Breast lumps need to be referred for further assessment because some of them may be due to cancer.

5. Which of the following is not a risk factor for breast cancer?

a. Cigarette smoking.

b. Excessive alcohol use.

c. Wearing a bra.

d. Family history of breast cancer.

e. Being overweight or obese.

Circle TRUE or FALSE for questions 6 through 10

6. TRUE or FALSE: Using any family planning causes breast cancer.

7. TRUE or FALSE: Breast cancer is curable if detected early.

8. TRUE or FALSE: Breast cancer can be prevented by living a healthy lifestyle.

9. TRUE or FALSE: Surgical treatment of breast cancer causes it to spread.

10. TRUE or FALSE: NHIF contributors can get cancer treatment paid for.

**Cervical Cancer**

Chose the one best answer:

1. What is cervical cancer?

a. cancer of the womb

b. Cancer of the lower abdomen

c. Cancer of the opening of the womb.

d. Any cancer of the woman’s reproductive organs.

e. None of the above

2. How is cervical cancer screened for?

a. By a clinician staining and observing the cervix.

b. By doing a urine test.

c. By doing a blood test for cancer cells.

d. By having an operation of the womb.

e. By feeling the lower abdomen.

3. Who should be screened for cervical cancer?

a. Only women above the age of 40years.

b. Women who have stopped having their periods.

c. All women who are sexually active.

d. Men whose wives have cervical cancer

e. Only people with family history of cervical cancer.

4. How can a person reduce their risk of getting cervical cancer?

a. By having one sexual partner.

b. By routinely attending cervical cancer screening.

c. By stopping cigarette smoking.

d. By reducing weight if overweight.

e. All of the above.

5. Which of the following is correct about cervical cancer treatment?

a. Cervical cancer always leads to death.

b. If picked early, cervical cancer can be cured.

c. Cervical cancer cure is achieved by taking herbal medicine.

d. Treatment can only be done in very specialized big hospitals.

e. NHIF does not cover treatment of cervical cancer.

6. TRUE or FALSE: Cervical cancer is caused by a familial curse.

7. TRUE or FALSE: surgery for cervical cancer causes rapid spread of the disease.

8. TRUE or FALSE: Regular screening for cervical cancer is the best way of preventing it.

9. TRUE or FALSE: Cervical cancer is mostly caused by a viral infection that’s sexually transmitted.

10. TRUE or FALSE: HIV infection increases the risk of cervical cancer.

**Section B. Clinician Written Test**

Choose the ONE best answer

1. The following are examples of Non Communicable diseases EXCEPT

a. Cancers

b. Cardiovascular disease

c. Chronic HIV infection

d. Chronic Obstructive pulmonary disease

2. In the diagnosis of diabetes mellitus;

a. All adults aged > 18 years are all type 2 diabetes patients

b. Children under one year can never have diabetes

c. A Random blood sugar alone is diagnostic

d. A fasting blood sugar is diagnostic

3. Mr. Bonde comes to your facility with a blood pressure reading of 130/121 mmHg from a community screening. You repeat the blood pressure and find that its 134/118 mmHg. What do you do for Mr. Bonde?

a. Educate him on hypertension and discharge him home since his blood pressure is normal and review him in 3 months

b. Discharge him home to repeat blood pressure measurement in 3 months

c. Educate him on hypertension and start on treatment as his blood pressure is elevated.

d. Admit him for close observation as his blood pressure is not stable and keeps changing.

4. Drug classes used in the management of hypertension include one of the following

a. Β- agonists

b. α – agonists

c. Angiotensin receptor blockers

d. Calcium channel agonists

5. Common side effects of antihypertensive drugs include the following EXCEPT

a. Hypokalemia- enalapril

b. Hyperuricemia– HCTZ

c. Angioedema- enalapril

d. Headache- Nifedipine

6. A fasting blood sugar of 8 mmol/L

a. Is normal

b. Is highly suspicious of diabetes

c. Is diagnostic of diabetes

d. Is diagnostic for pre diabetes

7. Which of the following patients has good glycemic control?

a. Mrs. A with a fasting blood sugar of 10.2mmol/L

b. Mrs. B with a HbA1c of 7.2%

c. Mrs. C with a random blood sugar of 12 mmol/L

d. Mrs. D with HbA1c of 10.2%

8. In differentiating type 2 and type 1 diabetes

a. Type 1 diabetes is more common in overweight and obese people while type 2 diabetes can affect anyone of any weight

b. Type 1 diabetes is more common in the elderly than in middle aged people

c. Type 2 diabetes is characterized by very elevated blood sugar at diagnosis and presence of urine ketones

d. Type 2 diabetes is likely to present with a complication of diabetes

9. The following antihypertensives are safe for use in pregnancy except

a. HCTZ

b. Nifedipine

c. Aldomet

d. Labetalol

10. Which of the following represents a correct prescription for diabetes medication

a. Metformin 1000mg TDS

b. Glimepiride 10mg OD

c. Glimepiride 2mg OD

d. Pioglitazone 5mg OD

11. In the initial assessment of a patient

a. A BMI of 29 is considered normal

b. Waist circumference is only useful in women

c. A pulse rate of 48 beats / minute is normal

d. Blood pressure should be taken in both arms

12. Other causes of hypertension include the following EXCEPT

a. Hypothyroidism

b. Pregnancy

c. Kidney disease

d. Varicose veins

13. Samuel is a 16 year old boy with a BMI of 26kg/m2, he has just been diagnosed with diabetes based on elevated blood sugar. What is the drug of choice in his management?

a. Metformin + glibenclamide as his blood sugar level is very high

b. Glibenclamide alone as he is not overweight

c. Insulin + glibenclamide for effective lowering of blood sugar level

d. Daily subcutaneous insulin

14. In the diagnosis of chronic complications of diabetes, which of the following is true?

a. Diabetes nephropathy can be diagnosed using a monofilament test

b. Diabetes retinopathy can be diagnosed using the snellen chart

c. Diabetes neuropathy can be diagnosed using a monofilament test

d. Diabetes induced cataracts can only be diagnosed by an ophthalmologist

15. The commonest available insulin is labeled 70/30. What does this mean?

e. It contains 70% of a short acting insulin and 30% of a long acting insulin

f. It contains 70 units of a short acting insulin and 30 units of an intermediate acting insulin

g. It contains 30 % of a short acting insulin and 70% of an intermediate acting insulin

h. It contains 30 units of a short acting insulin and 70 units of a long acting insulin

16. Mrs. Kenneth has been on follow up in your facility for 3 months recently her to dose of metformin was increased from 500Mg BD 1000mg BD. She comes to you complaining of severe abdominal discomfort with nausea and vomiting. What is the correct course of management for Mrs. Kenneth?

a. Treat her for typhoid as her chances of infection are high due to her diabetes

b. Investigate her for other cause of symptoms and reduce the dose of metformin

c. Allow her 2 weeks to assess if symptoms subside and advise her on lifestyle modification

d. Rehydrate her, stop all her medication, discharge home and review her after 2 weeks

17. A patients with hypertension presenting with a blood pressure of 210/120 mmHg with symptoms of poor vision and some weakness;

a. Must be managed with rapid infusion of IV antihypertensive medication to rapidly lower the blood pressure

b. Should be managed as outpatient with oral medication

c. Should be managed in the ward with oral medication and boluses of IV medication when BP rises again

d. Should preferably be managed in a facility with ICU capacity

18. In patients with diabetes and hypertension;

a. The drug of choice for hypertension management is an ACE inhibitor

b. The drug of choice for hypertension management is a Thiazide

c. The drug of choice for management of diabetes is metformin only

d. Creatinine measurement is of limited value in their measurement

19. Which of the following tests is correctly matched to the organ being assessed

a. Fundoscopy – kidney function

b. Creatinine- liver function

c. eGFR- renal function

d. Monofilament- kidney function

20. A blood pressure of 138/112 is

a. Normal

b. Stage 1 hypertension

c. Stage 2 hypertension

d. Stage 3 hypertension

**Supplementary File 5. Patient Self-Report/Feedback Forms**

**Patient feedback**

1. What has been your experience receiving care at this health facility for chronic diseases like diabetes and hypertension?

*(Tick one that apply)*

[ ] Very good

[ ] Good

[ ] Poor

[ ] Very poor

2. How useful have the following services been to you?

*(Choose 1-4; 1-Not useful, 2-somehow useful, 3-useful, 4- Very useful)*

|  | Not useful (1) | Somehow useful (2) | Useful (3) | Very useful (4) |
| --- | --- | --- | --- | --- |
| Health education |  |  |  |  |
| Screening for diabetes |  |  |  |  |
| Screening for hypertension |  |  |  |  |
| Assessment of your weight |  |  |  |  |
| Screening for cancer |  |  |  |  |
| Consultation with the doctors |  |  |  |  |
| Medications |  |  |  |  |

3. What are the challenges you or your community members face when trying to get medical care for chronic diseases like diabetes and hypertension?

*(Tick all that apply, do not read out responses)*

[ ] No challenges

[ ] Long distance to clinics

[ ] Lack of transport

[ ] Long queues at the clinics

[ ] Late start of clinics

[ ] Unclear clinic processes

[ ] Rude clinicians

[ ] Hurried consultation

[ ] Lack of explanation of one’s diagnosis

[ ] Expensive consultation

[ ] Expensive lab investigations

[ ] Expensive drugs

[ ] Lack of drugs

[ ] Lack of lab tests

[ ] Too short bookings for revisits

[ ] Very long bookings for revisits

[ ] Demand for bribes

[ ] Others: __________________________

4. What are reasons that you think people might not adhere to the diet and medication regimens prescribed by their physicians? *(Tick all that apply, do not read out responses)*

[ ] Failure to understand instructions

[ ] Feeling like they are being forced

[ ] Unavailability of prescribed diet

[ ] Lack of money to buy enough drugs

[ ] Lack of prescribed drugs

[ ] Side effects of drugs

[ ] Fear of addiction to prescribed drugs

[ ] Preference of herbal treatment

[ ] Preference of faith healing

[ ] Forgetfulness

[ ] Assuming they are cured

[ ] Lack of trust in the healthcare provider

[ ] Loss of hope

[ ] Other: ___________________________

**Supplementary File 6. Semi Structured Interviews with Facility, Sub-county and County Leaders**

**Tool A. Semi Structured Interview with Health Facility In-Charges**

**Note to Interviewer:**

· Ensure respondent meets the requirements in the recruitment form

· Follow the instructions indicated for each question

· Select a location that ensures privacy and space for the respondent to speak freely

**Introduction:**

· Start by introducing yourself, thanking the respondent for volunteering to participate in the study, and presenting the end line study and objectives of the SSI.

· Read the informed consent statement outlining all aspects of the study. Share information about the use of the recorder and who will hear this information.

· Obtain informed consent from the respondent, including consent for audio recording (if consent is not provided, do not audio record - use field notes).

**Background & Demographic Characteristics:**

1. Health facility:

2. Gender:

3. Professional cadre/designation:

4. Number of years in current role:

| **RE-AIM Area of Enquiry** | **Key Questions** |
| --- | --- |
| **Introduction** | 1. Thank you so much for meeting with me today. Can you tell me a bit about your role in this health facility?  2. How aware are you of the PIC4C project? Describe PIC4C activities that you are familiar with |
| **Which settings and individuals are willing to embrace specific aspects of the PIC4C model?** | |
| **Adoption** | 3. Describe to me how you have observed trained staff offering care in the training area covered by PIC4C project  4. Describe your health facility’s use of the mentorship program. Is your facility able to run independent of PIC4C mentors? Why/why not?  5. How well has your health facility implemented NHIF? Explain your answer  6. How well has your health facility implemented PIC4C initiated IT systems? Explain your answer. What are the facilitators? Barriers?  7. How has your health facility implemented PIC4C driven MoH tools? (Describe each tool). Explain your answer  8. Describe any healthcare strategies at your health facility that have included any aspect of the PIC4C strategies for any of the 4 conditions. |
| **What is the likelihood of the sustainability of PIC4C interventions and/or results?** **To what extent will it become institutionalized?** | |
| **Maintenance** | 9. To what extent have PIC4C elements been integrated into your health facility programs? Can you give me some specific examples?  10. Have you changed how you deliver services for the four conditions following the training that you received? If yes, how? Can you please provide some examples? Probe for details.  11. Now I would like to discuss the mentorship program. Did you receive mentoring to help improve your health service delivery practices at your facility? If so, how would you describe the mentoring that you received?  Did you find the mentorship useful? Why or why not?  How, if at all, did the mentorship influence the way you deliver services for the four conditions?  12. Describe how your health facility has institutionalized the RFP. How is it being maintained?  13. Describe how your health facility has institutionalized the PIC4C driven IT systems. How are they being maintained?  14. Do you think that any of the changes you identified in how you deliver services will continue after the PIC4C project ends? Why or why not?  In your opinion, what would influence whether these changes continue? |

**Closing:**

**-**Ask the participant if they have anything else they would like to contribute in terms of the discussion held.

-Thank the participants for their time. Remind the participant that the information will remain confidential and that they can find out more or ask any questions by calling the number provided on the consent document.

**Tool B. Semi Structured Interview with County, Sub-county, and Opinion Leaders**

**Note to Interviewer:**

· Ensure respondent meets the requirements in the recruitment form

· Follow the instructions indicated for each question

· Select a location that ensures privacy and space for the respondent to speak freely

**Introduction:**

· Start by introducing yourself, thanking the respondent for volunteering to participate in the study, and presenting the end line study and objectives of the SSI.

· Read the informed consent statement outlining all aspects of the study. Share information about the use of the recorder and who will hear this information.

· Obtain informed consent from the respondent, including consent for audio recording (if consent is not provided, do not audio record - use field notes).

**Background & Demographic Characteristics:**

1. County/Sub-county:

2. Gender:

3. Professional cadre/designation:

4. Number of years in current role:

| **RE-AIM Area of Enquiry** | **Key Questions** |
| --- | --- |
| **Introduction** | 1. Thank you so much for meeting with me today. Can you tell me a bit about your role in the County/Sub-county?  2. How aware are you of the PIC4C project? Describe PIC4C activities that you are familiar with  3. Are you aware that the PIC4C is coming to an end? If yes, ***Probe*** to find out respondents' knowledge on when project activities will end |
| **Which settings and individuals are willing to embrace specific aspects of the PIC4C model?** | |
| **Adoption** | 4. Describe to me how you have observed MoH PIC4C trained staff offering care in the training area covered by PIC4C  5. Describe health facilities’ use of the mentorship program. What has facilitated some facilities to run independent of mentors?  6. How well have your health facilities implemented NHIF? Explain your answer  7. How have your health facilities implemented PIC4C initiated IT systems? Explain your answer  8. How have your health facilities implemented PIC4C driven MoH tools? (Describe each tool). Explain your answer  9. Describe any County strategies or budgets that have included PIC4C strategies for any of the 4 conditions.  10. Which kinds of physical and structural changes have taken place within facilities that are being supported by PIC4C? |
| **What is the likelihood of the sustainability of PIC4C interventions and/or results?** **To what extent will it become institutionalized?** | |
| **Maintenance** | 11. To what extent have PIC4C elements been integrated into your communities and health facilities? Can you give me some specific examples?  12. Describe how your health facilities have institutionalized the RFP. How is it being maintained?  13. Describe how your health facilities have institutionalized the PIC4C driven IT systems. How are they being maintained?  14. Has there been absorption of clinical staff from PIC4C into County healthcare? If yes, describe how it has been happening. If no, explain why not? Would they be absorbed at the end of the PIC4C project? How?  15. Are there considerations to institutionalize CHV payment? If yes, how will it be done?  16. Do you think any of these activities will continue after the PIC4C project ends? Why or why not? How would you describe level of willingness to continue with the PIC4C model? Would they be able to continue care after the PIC4C project ends? Explain your answer  17. Will any changes or results due to these interventions be sustained when the PIC4C project ends? Why or why not? *Please describe further.* |

**Closing:**

· Ask the participant if they have anything else they would like to contribute in terms of the discussion held.

· Thank the participants for their time. Remind the participant that the information will remain confidential and that they can find out more or ask any questions by calling the number provided on the consent document.

**Supplementary File 6: Focus Group Discussions (FGDs) with Community health volunteers (CHVs), Community health promoters (CHPs Clinicians and Clients**

**Tool A. Focus Group Discussion with CHPs & CHVs**

**Introduction:**

· Start by introducing yourself, thanking the respondent for volunteering to participate in the study, and presenting the end line study and objectives of the FGD.

· Prior to or after the FGD, ask participants to fill in the participant information form (provide assistance as required). During this time, snacks/beverages can be distributed.

· Conduct a short icebreaker to help participants feel comfortable.

**Participant information form (to be filled for each participant):**

1. Health facility:

2. Gender:

3. Professional cadre/designation:

4. Number of years in current role at this facility:

| **RE-AIM Area of Enquiry** | **Key Questions** |
| --- | --- |
| **Introduction** | 1. Thank you so much for meeting with us today. Can you tell me what you know about the PIC4C project? Describe PIC4C activities that you are familiar with. |
| **What is the impact of PIC4C programs on important outcomes?** | |
| **Effectiveness** | 2. Describe effects @ PIC4C interventions in building knowledge and skills among CHPs & CHVs to serve clients with the four conditions (*Discuss @ relevant intervention sequentially. Probe for changes in knowledge, skills, attitude, confidence)*  3. Describe effects @ PIC4C interventions in delivery of care at the health facilities (*Discuss @ relevant intervention sequentially. Probe for changes in supplies & equipment, affordability accessing services, access to drugs, # of staff, linkage treatment & retention of client)*  4. Generally, describe NHIF uptake among clients seen at the health facilities. How has the PIC4C project influenced NHIF uptake? Explain your thoughts.  5. In your opinion, what changes have you noticed in the quality of life of your patients who have been touched by the PIC4C interventions?  6. Describe any economic outcomes associated with the PIC4C project. Provide specific examples.  7. What can be reported as negative effects of PIC4C interventions in this area? (*Discuss @ relevant intervention sequentially. List all negative effects mentioned then discuss each of them in depth)* |
| **Which settings and individuals are willing to embrace specific aspects of the PIC4C model?** | |
| **Adoption** | 8. What is your opinion on trainings offered by PIC4C programs to CHPs & CHVs?  9. Describe your health facility’s use of the mentorship program.  How would you describe the mentoring that you received?  Did you find the mentorship useful? Why or why not?  Is your facility able to run independent of PIC4C mentors? Why/why not? |
| **What is the likelihood of the sustainability of PIC4C interventions and/or results?** **To what extent will it become institutionalized?** | |
| **Maintenance** | 10. How, if at all, did the mentorship program influence the way you deliver services for the four conditions?  11. Have you changed how you deliver services for the four conditions following the training that you received? If yes, how? Can you please provide some examples? Probe for details.  12. Do you think that any of the changes you identified in how you deliver services will continue after the PIC4C project ends? Why or why not?  In your opinion, what would influence whether these changes continue? |

**Closing:**

· Ask the participants if they have anything else they would like to contribute in terms of the discussion held.

· Thank the participants for their time.

**Tool B. Focus Group Discussion with Healthcare Providers**

**Introduction:**

· Start by introducing yourself, thanking the respondent for volunteering to participate in the study, and presenting the end line study and objectives of the FGD.

· Prior to or after the FGD, ask participants to fill in the participant information form (provide assistance as required). During this time, snacks/beverages can be distributed.

· Conduct a short icebreaker to help participants feel comfortable.

**Participant information form (to be filled for each participant):**

1. Health facility:

2. Gender:

3. Professional cadre/designation:

4. Number of years in current role at this facility:

| **RE-AIM Area of Enquiry** | **Key Questions** |
| --- | --- |
| **Introduction** | 1. Thank you so much for meeting with us today. Can you tell me what you know about the PIC4C project? Describe PIC4C activities that you are familiar with. |
| **What is the impact of PIC4C programs on important outcomes?** | |
| **Effectiveness** | 2. Describe effects @ PIC4C interventions in building knowledge and skills among health providers to serve clients with the four conditions (*Discuss @ relevant intervention sequentially. Probe for changes in knowledge, skills, attitude, confidence)*  3. Describe effects @ PIC4C interventions in delivery of care at the health facilities (*Discuss @ relevant intervention sequentially. Probe for changes in supplies & equipment, affordability accessing services, access to drugs, # of staff, linkage treatment & retention of client)*  4. Generally, describe NHIF uptake among clients seen at this facility. How has the PIC4C project influenced NHIF uptake? Explain your thoughts.  5. In your opinion, what changes have you noticed in the quality of life of your patients who have been touched by the PIC4C interventions?  6. Describe any economic outcomes associated with the PIC4C project. Provide specific examples.  7. What can be reported as negative effects of PIC4C interventions in this area? (*Discuss @ relevant intervention sequentially. List all negative effects mentioned then discuss each of them in depth)* |
| **Which settings and individuals are willing to embrace specific aspects of the PIC4C model?** | |
| **Adoption** | 8. What is your opinion on training offered by PIC4C programs to healthcare providers?  9. Describe your health facility’s use of the mentorship program.  How would you describe the mentoring that you received?  Did you find the mentorship useful? Why or why not?  Is your facility able to run independent of PIC4C mentors? Why/why not?  10. How well has your health facility implemented NHIF? Explain your answer  11. How well has your health facility implemented PIC4C initiated IT systems? Explain your answer. What are the facilitators? Barriers?  12. How has your health facility implemented PIC4C driven MoH tools? (Describe each tool). Explain your answer  13. What is the likelihood that the PIC4C model or specific aspects of it will be sustained in your health facility following the end of the project? Explain your answer |
| **What is the likelihood of the sustainability of PIC4C interventions and/or results?** **To what extend will it become institutionalized?** | |
| **Maintenance** | 14. How, if at all, did the mentorship program influence the way you deliver services for the four conditions?  15. To what extent have PIC4C elements been integrated into your health facility programs? Can you give me some specific examples? (*Discuss each element separately*)  16. Have you changed how you deliver services for the four conditions following the training that you received? If yes, how? Can you please provide some examples? Probe for details.  17. Describe how your health facility has institutionalized the RFP. How is it being maintained?  18. Describe how your health facility has institutionalized the PIC4C driven IT systems. How are they being maintained?  19. Do you think that any of the changes you identified in how you deliver services will continue after the PIC4C project ends? Why or why not?  In your opinion, what would influence whether these changes continue? |

**Closing:**

· Ask the participants if they have anything else they would like to contribute in terms of the discussion held.

· Thank the participants for their time

**Tool C. Focus Group Discussion with Clients (Hypertension, Diabetes, Breast and Cervical Cancers)**

**Introduction:**

· Start by introducing yourself, thanking the respondent for volunteering to participate in the study, and presenting the end line study and objectives of the FGD.

· Prior to or after the FGD, ask participants to fill in the participant information form (provide assistance as required). During this time, snacks/beverages can be distributed.

· Conduct a short icebreaker to help participants feel comfortable.

**Participant information form (to be filled for each participant):**

1. Gender:

2. Health condition (Chronic disease):

3. Education level:

4. Village/Settlement/Sub Location:

5. Nearest public facility offering their care for the chronic disease:

| **RE-AIM Area of Enquiry** | **Key Questions** |
| --- | --- |
| **Introduction** | 1. Thank you so much for meeting with us today. Can you tell me what you know about the PIC4C project? Describe PIC4C activities that you are familiar with. |
| **What is the impact of PIC4C programs on important outcomes?** | |
| **Effectiveness** | 2. Describe effects @ PIC4C interventions in knowledge among clients and the general community members on the four conditions (*Discuss @ relevant intervention sequentially. Probe for changes in knowledge, attitudes to screening treatment and adherence)*  3. Describe effects @ PIC4C interventions in delivery of care at the health facilities (*Discuss @ relevant intervention sequentially. Probe for changes in supplies & equipment, affordability accessing services, access to drugs, # of staff, attitude of healthcare providers, linkage treatment & retention of clients, clients’ attitudes to services offered at the health facilities)*  4. How has clients’ care seeking behavior for the four conditions changed since the coming of the PIC4C programs? What are their experiences? (*Probe for reports about all four conditions. Probe for both positive and negative experiences*)  5. Generally, describe NHIF uptake among people with the four conditions. How has the PIC4C project influenced NHIF uptake? Explain your answers.  6. In your opinion, what changes have you noticed in the quality of life of patients who have been touched by the PIC4C interventions? Provide specific examples  7. Describe any economic outcomes associated with the PIC4C project. Provide specific examples.  8. What can be reported as negative effects of PIC4C interventions in this area? (*Discuss @ relevant intervention sequentially. List all negative effects mentioned then discuss each of them in depth)* |

**Closing:**

· Ask the participants if they have anything else they would like to contribute in terms of the discussion held.

· Thank the participants for their time.
